# Efficacy of platelet-rich plasma in arthroscopic repair for discoid lateral meniscus tears

**DOI:** 10.1186/s12891-019-2500-9

**Published:** 2019-03-18

**Authors:** Wen-Li Dai, Hua Zhang, Ze-Ming Lin, Zhan-Jun Shi, Jian Wang

**Affiliations:** 1 Department of Orthopedic Surgery, Nanfang Hospital, Southern Medical University, 1838 Guangzhou Road, Guangzhou, 510515 China; 2 Department of Orthopaedics, The First Affiliated Hospital of Chongqing Medical University, 1 Yixueyuan Road, Chongqing, 400016 China

## Abstract

**Background:**

To evaluate the clinical results of arthroscopic repair with or without platelet-rich plasma (PRP) for tears of the discoid lateral meniscus (DLM).

**Methods:**

Twenty-nine patients with DLM tears within a stable knee were arthroscopically treated with meniscal suture repair. Of those, 14 were augmented with platelet-rich plasma (PRP), and 15 were performed without PRP augmentation. Patients were evaluated at baseline (the day before surgery) and then 12 and 24 months after the last injection. Evaluation included the Lysholm score, and Ikeuchi grade, Visual analogue score (VAS) for pain and failure rate. Failure was defined by patients developing symptoms of joint line pain, locking, swelling or requiring repeat arthroscopy.

**Results:**

There was no difference in the failure rate in the PRP group (1 of 14) compared with the non-PRP group (2 of 15) (*P* = 0.58). Statistically significant improvement in Lysholm score, Ikeuchi grade and VAS for pain was documented at the last follow-up compared with baseline in both PRP and non-PRP group. No significantly difference was found between the PRP group and non-PRP group on Lysholm score, Ikeuchi grade and VAS for pain at the last follow-up. In the univariate analysis of each variable, younger age (*P* = 0.036) and longer follow-up duration (*P* = 0.043) were statistically associated with a better function improvement. Whereas in multivariate analysis, only younger age (*P* = 0.004) was significantly associated with a better function improvement.

**Conclusion:**

With regard to clinical evaluations in arthroscopic repair for DLM tears, PRP group had similar effect in pain relief and functional improvement to non-PRP group at mid-term follow-up. Future larger prospective studies with a longer follow-up are needed to determine whether PRP should be used with DLM repair.

## Background

Discoid lateral meniscus (DLM) is an abnormal variation of meniscus which was first described by Young in 1889 in a cadaver specimen. The reported prevalence of DLM ranges from 0.4 to 17% [1–3]. It is rare in Caucasian, but more common in Asian [2, 4]. Because these menisci are larger and thicker than normal lateral meniscus, DLM is associated with a higher frequency of meniscal tears and related symptoms [3, 5]. Many DLM cases have an associated tear in the DLM, resulting in symptoms such as such as pain, snapping, swelling, buckling and locking, and surgery is often considered when conservative methods of treatment [2, 6, 7].

Traditionally, total meniscectomy has been thought that it could provide a good short-term outcome for DLM patients [1, 8], but recently the advantages and the improvement that arthroscopy has offered widened its application and permitted more accurate diagnosis and treatment of the lesion [6, 9]. Recent biomechanical studies of knee function have revealed the importance of the menisci, and meniscus-preserving procedures (partial meniscectomy with or without repair), instead of total meniscectomy, for a torn DLM have been advised [10–12]. To date, meniscal repairs have been extensively studied but continue to fail for varied reasons [11, 13]. It is thought that the lack of vasculature providing intrinsic nutrition is one reason for poor healing and may explain the higher success rate of meniscal repair in concomitant anterior cruciate ligament (ACL) reconstruction [14].

Platelet-rich plasma (PRP) is an autologous blood product that contains increased concentrations of cytokines including vascular endothelial growth factor, transforming growth factor-b, epidermal growth factor, fibroblast growth factor, platelet-derived growth factor. The various cytokines in PRP are known to positively affect fibrochondrocyte migration and extracellular matrix production in vitro [15–17]. Ishida et al. compared the effect of PRP with platelet poor plasma on meniscal tissue and found significant positive effects of PRP on cell viability/proliferation and matrix production [18]. Furthermore, Howard et al. found PRP was able to increase meniscal cell number above peripheral whole blood and up-regulated gene expression of Aggrecan, Collagen type I, and Elastin [19]. However, Freymann et al. evaluate the migratory, proliferating, and extracellular matrix forming effect of PRP on meniscus cells and found PRP showed no inducing effect on aggrecan and cartilage oligomeric matrix protein [20].To the best of our knowledge, no studies have investigated clinical outcomes of arthroscopic repair with PRP augmentation for DLM tears.

Therefore, the purpose of the current study was to evaluate the clinical results of arthroscopic repair with or without PRP for tears of the DLM. We hypothesized that arthroscopic repair for DLM tears with PRP would lead to improvements in function and pain outcomes due to the release of bioactive molecules that would possibly affect the DLM healing.

## Methods

### Patients

**Fig. 1:**
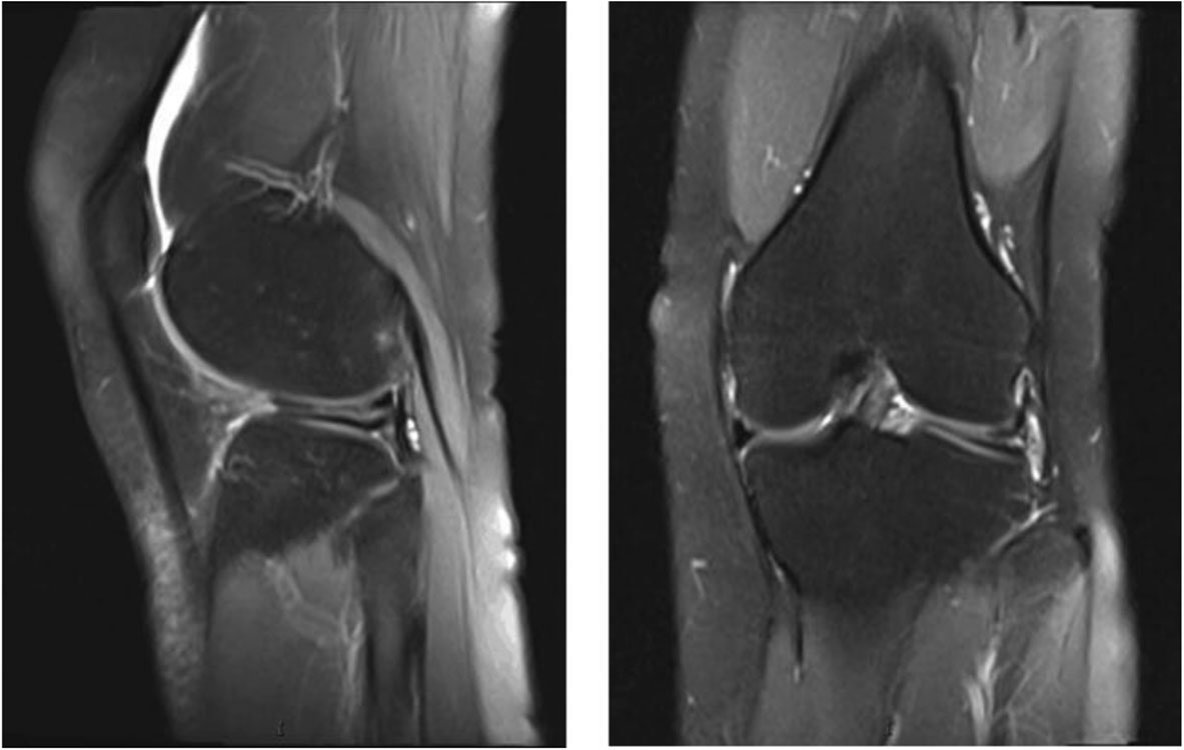
MRI demonstrates a horizontal discoid lateral meniscus tear

From July 2013 and October 2015, the medical records of 53 patients who had undergone arthroscopic surgery for symptomatic DLM by an experienced surgeon (J.W) were retrospectively reviewed. Of those, 32 DLM patients in whom we performed an arthroscopic repair were identified. Inclusion criteria were as follows: no former ipsilateral meniscus surgery, no commitment surgery such as anterior cruciate ligament reconstruction, and a meniscal tear size > 10 mm. The exclusion criteria included patients did not underwent arthroscopic repair of torn of DLM and age older than 60 years. Of those patients, 16 were augmented with platelet-rich plasma (PRP), and 16 were performed without PRP augmentation. All 32 patients had radiographic evidence of meniscal pathology seen on magnetic resonance imaging (MRI) (Fig. 1). Despite our efforts, 3 of the selected patients were lost to follow-up due to migration (2 patients in the PRP group and 1 in the non-PRP group). We evaluated the remaining 29 patients, 14 in the PRP group and 15 in the non-PRP group. No second-look imaging or second-look arthroscopy was performed in this study.

### PRP preparation

To obtain the PRP, 37 ml of the patient’s blood was collected into a 50-ml injector containing 4 ml 3.8% sodium citrate as anticoagulant. Then, 2 centrifugations were performed: the first at 2000 rpm for 10 min to separate erythrocytes, and the second also at 2000 rpm for 10 min to concentrate platelets, which provided 4 ml of PRP. The preparation method used allowed the number of platelets per milliliter to increase by a mean of 6.4 ± 1.6 times (range, 3.5–8.7) with respect to baseline blood values. Injected PRP in this study also contained leukocytes (leukocyte-rich PRP) 6.1 ± 1.5 times (range, 3.1–8.4) times with respect to the normal blood value.

### Operative procedure

**Fig. 2:**
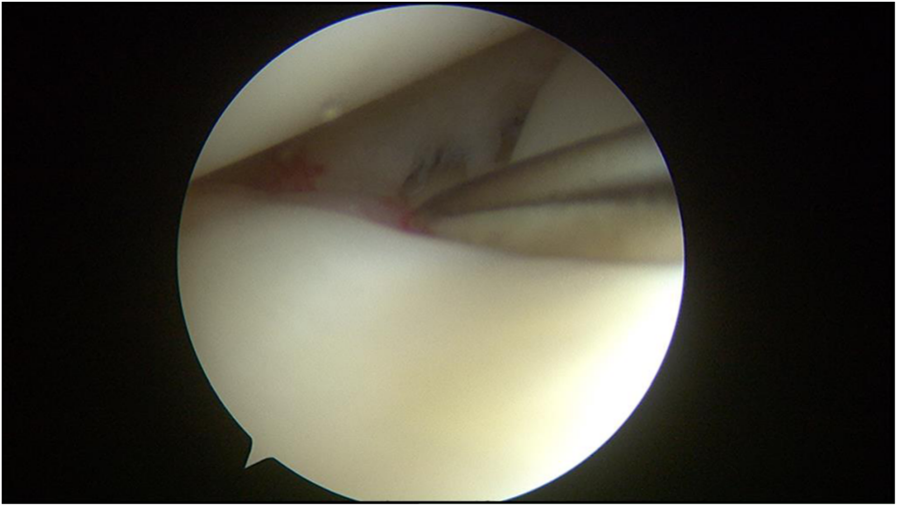
A hook probe was used to confirm a meniscal tear in DLM

**Fig. 3:**
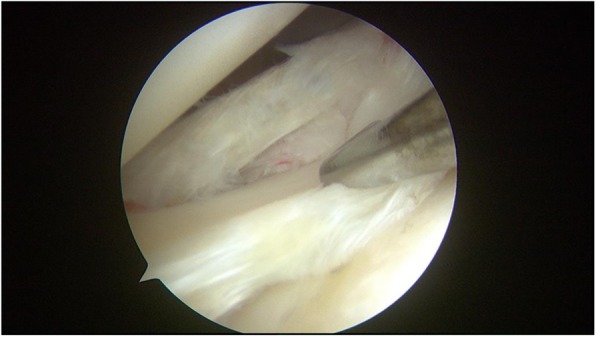
Saucerization was performed for DLM and meniscal instability was again confirmed

**Fig. 4:**
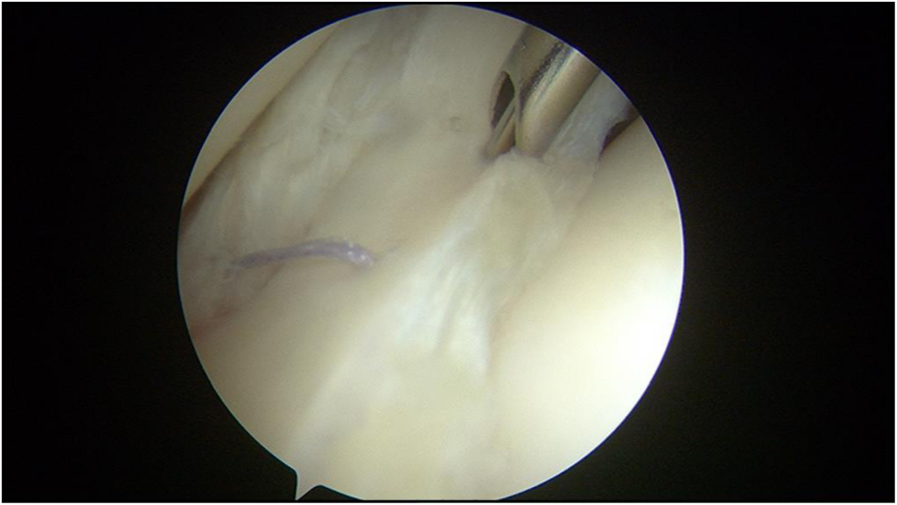
The meniscus repair was performed using the inside-out technique

The meniscus repair was performed using the inside-out technique. Repair was only performed in the red-red zone or red-white zone of the posterior horn of the medial or lateral meniscus, which is reported to have good healing potential [21]. An arthroscopic examination was performed via anteromedial and anterolateral portals. A hook probe (Smith & Nephew, Andover, MA) was used to confirm a lateral meniscal tear (Fig. 2). Once the tear pattern was confirmed, an arthroscopic punch (Smith & Nephew, Andover, MA) and shaver (Smith & Nephew, Andover, MA) were used to perform saucerization of the discoid meniscus. Meniscal instability was again confirmed, and preparation for repair was undertaken (Fig. 3). The torn margin of the meniscus and adjacent synovium were abraded with a rasp and shaver to improve the vascular supply to the lesion. A medium Graves speculum blade (MedGyn, Addison, IL) was then positioned as a meniscal retractor to aid in retrieving sutures and protecting the neurovascular structures behind the knee by an incision parallel and just posterior to the lateral collateral ligament. Once the retractor was in place, two double-armed needles with 2–0 polyester braided suture (Ethicon, Somerville, NJ) were placed vertically every 4 to 6 mm and were tied to appose the meniscus body to the remaining meniscus rim and attachment using a zone-specific cannula (Linvatec, Largo, FL) (Fig. 4).

**Fig. 5:**
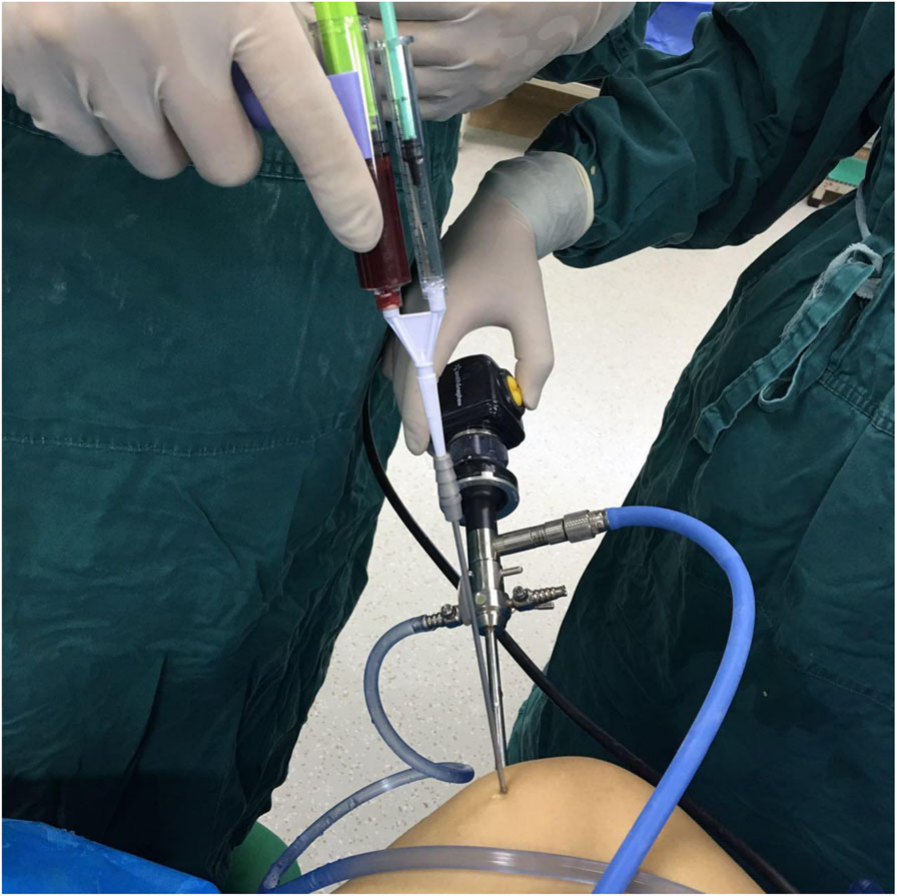
PRP and thrombin were injected simultaneously on the repaired site under arthroscopic vision

**Fig. 6:**
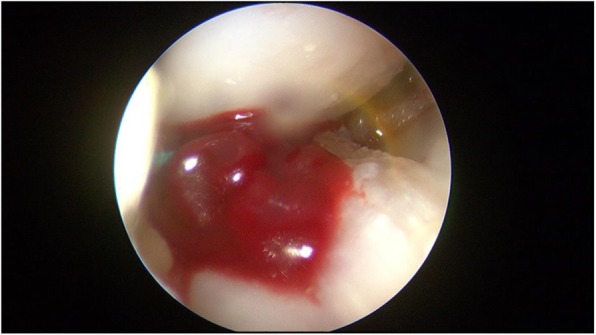
After the PRP gel clot was formed on the repair site, the arthroscope was pulled out

After the repair, excess saline was suctioned out. Then, PRP (4 ml) and 500 IU thrombin (1 ml) were injected simultaneously on the repaired site using two injectors (one 5-ml for PRP and the other 1-ml for thrombin) and a cannula needle 2.5 mm in diameter under arthroscopic vision (Fig. 5). The meniscal sutures previously placed were loosened so that the PRP can have the best contact area with the lesion. After the PRP gel clot was formed on the lesion (Fig. 6), the knee was taken to 90° of flexion and the sutures were fastened down and then tied. Finally, the arthroscope was pulled out, and the portals were then sutured. No drainage was used after the surgery.

### Rehabilitation protocol

In this study, all patients followed the same rehabilitation protocol. For the first 2 weeks, patients were allowed to bear partial body weight up to 10 kg, with range of motion from 0° to 60°. In the third and fourth weeks, partial weight-bearing of 50% of body weight was permitted, with range of motion from 0° to 90°, After the fourth week, unassisted walking with full weight-bearing was permitted and full joint movement exercises were started. No squatting or deep flexion activities greater than 120° were permitted for 3 months, and running, jumping, and cutting were restricted for 6 months.

### Outcome assessment

Patients were evaluated at baseline (the day before surgery) and then 12 and 24 months after the last injection; evaluation included the Lysholm score [22], and Ikeuchi grade [2], Visual analogue score (VAS) for pain [23] and failure rate. Failure was defined by patients developing symptoms of joint line pain and/or locking or swelling or requiring repeat arthroscopy. Outcome data were collected in person or by telephone by an orthopaedic surgeon who was blinded to the treatment received by the patient.

### Statistical analyses

All data management and statistical analysis were performed with SPSS version 22.0 software (SPSS Inc., Chicago, IL, USA). Student’s t-tests were used for continuous variables (age, symptom duration, duration of follow-up, Lysholm score and VAS for pain) and Fisher exact test was used to analyze the categorical variables (gender, type of DLM, repaired meniscal zone, Ikeuchi grade and failure rate). In our study, the failure rate was demonstrated by intent-to-treat (ITT) and per-protocol (PP) analyses. In the ITT approach, all patients were included in the analysis in the group to which they were allocated regardless of loss to follow-up. In the PP approach, patients who completed the entire procedure were included in the analysis. The level of significance was set at *P* < 0.05.

We conducted univariate linear regression analysis to identify variables associated with pain relief and function improvement. Then we performed multivariate linear regression analysis including variables with a *P* value < 0.05 defined by univariate analysis.

For this study, the sample size calculation for patients was done according to the previous study by Pujol and colleagues [24]. Our hypothesis was that there would be a 6% relative difference in failure rate between the PRP and non-PRP group, which meant that a sample size of 349 patients in each group was needed to get a power of 80% for a significance level of 5%.

## Results

### Characteristics of patients

There were 6 male and 8 female patients in PRP group, 5 male and 10 female patients in non-PRP group. Among the 29 patients, 14 were injured on the left side and 15 on the right. Among the patients, the most frequent lesion was longitudinal tears, which was found in 11 knees, and complex tears were found in 10 knees, horizontal cleavage in 7 knees, and radial tears in 1 knee. 21 patients had the repair in the red zone and 8 in the red-white zone. The mean length of follow-up was 20.6 months (range 12–27 months). Of these patients, 6 patients in PRP group and 6 patients in non-PRP group had a follow-up greater than 24 months. The mean age at the time of surgery was 32.4 years (range, 13 to 52 years) in PRP group, 30.3 years (range, 14 to 50 years) in non-PRP group. According to Watanabe’s classification, 17 knees were classified as type 1 (complete type) and 12 knees were classified as type 2 (incomplete type); there were no type 3 (Wrisberg ligament type).

### Lysholm score

In the PRP group, the mean Lysholm knee score improved from 53.3 ± 12.7 to 79.8 ± 9.6 at the last follow-up (*P* < 0.0001). In the non-PRP group, the mean Lysholm knee score improved from 55.0 ± 9.3 to 74.6 ± 11.6 at the last follow-up (*P* < 0.0001). However, no significantly difference was found between the PRP group and non-PRP group on Lysholm score at the last follow-up (*P* = 0.306).

Table 1 summarizes the factors associated with the Lysholm score on univariate analyses. Results showed patients with younger age (*P* = 0.036) and longer follow-up duration (*P* = 0.043) were associated with a better function improvement (Lysholm score). Whereas in multivariate analysis, only younger age (*P* = 0.004) was significantly associated with a better function improvement after the surgery (*P* = 0.080 for the duration of follow-up).

**Table 1:** Factors associated with the Lysholm score on univariate analyses

Characteristic	Lysholm score		
	Number of patients	Lysholm score	*P* Value
Age, y			
Age ≤ 30	14	80.7 ± 12.9	0.036
Age > 30	15	72.7 ± 5.4	
Gender			
Male	11	76.7 ± 9.4	0.948
Female	18	76.4 ± 12.4	
Symptom duration, m			
Duration ≤3	10	74.7 ± 9.0	0.680
Duration > 3	19	77.6 ± 11.2	
Type of DLM			
Complete DLM	17	78.1 ± 10.3	0.368
Incomplete DLM	12	74.5 ± 10.6	
Repaired meniscal zone			
R-R	21	75.2 ± 8.7	0.256
W-R	8	80.2 ± 14	
Duration of follow-up, m			
Duration < 24	17	73.3 ± 9.2	0.043
Duration ≥24	12	81.2 ± 10.6	

### VAS for pain

In the PRP group, the mean VAS score decreased from 4.1 ± 1.0 to 1.2 ± 1.0 at the last follow-up (*P* < 0.0001). In the non-PRP group, the mean VAS score decreased from 3.4 ± 1.3 to 1.6 ± 1.1 at the last follow-up (*P* < 0.0001). However, no significantly difference was found between the PRP group and non-PRP group at the last follow-up (*P* = 0.321).

Table 2 summarizes the factors associated with the VAS score on univariate analyses. Results showed factors including age, gender, symptom duration, type of DLM, duration of follow-up and repaired meniscal zone were not associated with a better pain relief after the surgery.

**Table 2:** Factors associated with the VAS score on univariate analyses

Characteristic	VAS for pain		
	Number of patients	VAS for pain,	*P* Value
Age, y			
Age ≤ 30	14	1.1 ± 1.1	0.169
Age > 30	15	1.7 ± 0.9	
Gender			
Male	11	1.2 ± 1.2	0.436
Female	18	1.5 ± 0.9	
Symptom duration, m			
Duration ≤3	10	1.4 ± 1.2	0.835
Duration > 3	19	1.4 ± 1.0	
Type of DLM			
Complete DLM	17	1.4 ± 1.1	0.993
Incomplete DLM	12	1.4 ± 1.0	
Repaired meniscal zone			
R-R	21	1.6 ± 1.1	0.079
W-R	8	0.8 ± 0.7	
Duration of follow-up, m			
Duration < 24	17	1.6 ± 1.0	0.158
Duration ≥24	12	1.0 ± 1.0	

### Ikeuchi grade

In the PRP group, none of the 14 knees (0.0%) showed clinically excellent or good results at baseline, whereas 10 of 14 (71.4%) were documented with excellent or good results at the last follow-up (*P* < 0.0001). In the non-PRP group, none of the 15 knees (0.0%) showed clinically excellent or good results at baseline, whereas 12 of 15 (80.0%) were documented with excellent or good results at the last follow-up (*P* < 0.0001).

Compared the baseline, statistically significant improvement was found both in PRP group and non-PRP group at the last follow-up. However, there was no significant difference in Ikeuchi grade between the PRP group and non-PRP group at the last follow-up (*P* = 0.601).

Table 3 summarizes the factors associated with the Ikeuchi grade on univariate analyses. Results showed factors including age, gender, symptom duration, type of DLM, duration of follow-up and repaired meniscal zone were not associated with a better function improvement (Ikeuchi grade) after the surgery.

**Table 3:** Factors associated with the Ikeuchi grade on univariate analyses

Characteristic	Ikeuchi grade				
	Excellent (*n* = 11)	Good (*n* = 11)	Fair ((*n* = 7)	Poor (*n* = 0)	*P* Value
Age, y	30.3 ± 16.8	28.8 ± 13.8	36.8 ± 13.7	–	0.247
Gender (Male/Female), n	5/6	4/7	2/5	–	0.765
Symptom duration, m	14.2 ± 18.8	16.0 ± 17.8	14.1 ± 11.3	–	0.819
Type of DLM (complete/incomplete), n	8/3	4/7	5/2	–	0.163
Repaired meniscal zone (R-R/W-R), n	7/4	7/4	7/0	–	0.172
Duration of follow-up, m	22.6 ± 4.5	17.9 ± 4.6	21.8 ± 4.5		0.098

### Failure rate

The failure rate in our study was 10.3% (3 of 29 patients) at a mean of 20.6 years postoperatively, with 1 patients in the PRP group and 2 in the non-PRP group. There were no significant difference in the failure rate between PRP and non-PRP groups in both ITT (*P* = 0.63) and PP (*P* = 0.58) analyses. The average time from surgery to failure of meniscal repair was 22.7 months. For these 3 patients, 1 patient sustained a new injury to the operative knee. If this patient is excluded, our atraumatic failure rate of meniscal repair is 6.9%.

## Discussion

The purpose of the current study is to evaluate the clinical results of arthroscopic repair with or without PRP for tears of the DLM. The results of this study show that arthroscopic repair with PRP augmentation had similar effect in pain relief, functional improvement and failure rate to non-PRP group for DLM patients at mid-term follow-up.

The application of PRP was developed based on studies demonstrating the physiological roles of several bioactive proteins expressed in platelets, which lead to tissue regeneration [25]. Many in vitro studies have demonstrated that injection of various growth factors could stimulate repair of the meniscus tissue [26–28]. Platelet-derived growth factor (PDGF) has mostly been evaluated in sheep menisci. Following the use of PDGF, cell proliferation and migration and extracellular collagen matrix formation were increased in torn meniscus zones when compared to the control [15, 28, 29]. In the study of Cole et al., the value of PRP use in meniscal repair is the possibility of delivering a local concentration of growth factors and other cytokines directly to the repair site [30].

However, despite the promising preclinical findings, the use of PRP remains controversial in meniscal repair. In a study of arthroscopic meniscal repair [31], Griffin et al. reported that there was no difference in the proportion of patients who underwent reoperation in the PRP group (27%) compared with the non-PRP group (25%, *P* = 0.89). Functional outcome measures were not different between the two groups (*P* = 0.55). Furthermore, there was also no difference in the proportion of patients who returned to their regular sports/activities in the PRP group (71%) compared with the non-PRP group (78%, *P* = 0.75). Whereas in a study of open meniscal repair [24], Pujol et al. reported that the difference between PRP and non-PRP augment groups was significant for pain and sports activities parameters in KOOS score (*P* = 0.046 and 0.03, respectively). Furthermore, there was a statistically significant difference in the healing appearance of repaired menisci by MRI evaluation between the PRP and non-PRP groups (*P* < 0.01).

To the best of our knowledge, no studies concerning the clinical effects of PRP on meniscal repair for torn DLM have been published to date. We therefore sought to evaluate whether PRP augmentation during arthroscopic repair decreased the rate of subsequent meniscectomy, whether PRP augmentation affected validated functional and pain outcome scores, and whether the outcomes differed by the age, gender, type of DLM, symptom duration, repaired meniscal zone. We found that there was no difference in pain relief, functional improvement and failure rate between patients with and without PRP augmentation and only younger age was significantly associated with a better function improvement (Lysholm score) in multivariate analysis (*P* = 0.004). Gender, symptom duration, type of DLM, and repaired meniscal zone were not associated with a better function improvement (Lysholm score, Ikeuchi grade) and pain relief (VAS for pain) after the surgery.

There are some tips on DLM tears repair based on our experience. Firstly, the tear should be identified and characterized based on its size, location, and overall quality before the repair. Secondly, the tear should be anatomically reduced and the sutures should be placed perpendicularly to the lesion to restore its anatomic position. In addition, we prefer the inside-out repair because of the ability to confer greater stability to the lesion via increased number of sutures, and not having to use a large intra-articular device that allows for greater versatility.

This study had several limitations, including the small number of patients, the retrospective design of the study, and the lack of long-term follow-up. The second limitation was that there is no objective measurement of clinical outcome such as postoperative magnetic resonance imaging or second-look arthroscopy to evaluate the consistency of the repair. If second-look arthroscopy had been performed, the failure rate could conceivably be even higher.

## Conclusion

With regard to clinical evaluations in arthroscopic repair for lateral discoid meniscus tears, PRP group had similar effect in pain relief and functional improvement to non-PRP group at mid-term follow-up. Future larger prospective studies with a longer follow-up are needed to determine whether PRP should be used with DLM repair.

## Abbreviations

ACL: Anterior cruciate ligament
DLM: Discoid lateral meniscus
MRI: Magnetic resonance imaging
PDGF: Platelet-derived growth factor
PRP: Platelet-rich plasma
VAS: Visual analogue score
